# Relationship of Performance Measures and Muscle Activity between a 180° Change of Direction Task and Different Countermovement Jumps

**DOI:** 10.3390/sports8040047

**Published:** 2020-04-10

**Authors:** Hallvard Nygaard Falch, Håvard Guldteig Rædergård, Roland Van den Tillaar

**Affiliations:** Department of Sport Sciences and Physical Education, Nord University, 7600 Levanger, Norway; falch7@hotmail.com (H.N.F.); hovi_7@hotmail.com (H.G.R.)

**Keywords:** electromyography, countermovement jumps, specificity, horizontal uni- and bilateral jumps

## Abstract

The ability to rapidly perform change of direction (COD) is crucial for performance in Soccer. COD speed is thought to share similarities with countermovement jumps in kinematics and muscle activation. Thus, the objective of the current study was to investigate the relationship between muscle activities in performance measures of a modified 505-agility test and different countermovement jumps. Twenty-one experienced soccer players performed a COD test including the 505-agility test and uni- and bi-lateral horizontal and vertical countermovement jumps. The main findings were that the vertical bilateral and horizontal unilateral countermovement jump were able to predict total time to complete the COD, but not 505-agility time. Muscle activity in the COD and countermovement jumps was only distinguished by a higher peak muscle activity for the adductor longus, semitendinosus and biceps femoris in the COD to stabilize the hip and decelerate knee joint movements when turning compared with the jumps. Conclusively, the relationship between performance in countermovement jumps and total time to complete the COD test was due to longer sprint distances, which makes the distinction between performances bigger. Peak muscle activity of most muscles is similar between the jumps and the COD step, indicating similar muscular demands between these activities.

## 1. Introduction

Soccer is an intermittent sport [1], where high-intensity actions of short duration (<5 s) occur frequently throughout a match [2,3,4], such as jumps, sprints, tackles, accelerations and rapid changes of direction [5], often referred to as ‘agility’. High intensity actions predominantly require high energy phosphates as phosphocreatine, which is utilized anaerobically [6]. The athletes’ aerobic capacity (i.e., maximal oxygen consumption) is also important in this context by determining the ability to repeat these actions, as it enables high energy phosphates to be partly or fully restored [7]. This demands soccer players to training upon these high-intensity actions.

One of these actions is agility, defined in earlier research as a rapid whole-body movement in response to a stimuli [8], consisting of both technical, cognitive and physical aspects [9]. Strength and conditioning coaches often seek to improve the physical aspect of agility, which in research terminology is known as developing the change of direction (COD) ability [10]. The COD consists of an acceleration, before decelerating caused by eccentric muscle work and then rapidly changing momentum into a new direction by producing a rapid concentric-propulsive force [11]. Improving the physical factors influencing COD performance may lead to an overall greater performance in competition settings as soccer players are found to turn ≈700 times per game [12]. Greater COD abilities helps getting a physical and tactical advantage as goals often are preceded CODs, [13] by surpassing opponents or creating space. As such, more knowledge of how to improve the COD performance is of great importance to sport and conditioning coaches. Several studies have utilized strength training [14,15,16,17,18,19,20], specific COD drills [21,22,23,24,25], plyometric training [26,27,28,29] and a combination of these different training forms [30,31,32,33,34,35] to the physical aspect of COD performance [9]. 

Plyometric training is thought to share similarities with COD due to the objective of exerting maximal amount of force in a restricted time period, producing as much power as possible [36]. In addition, utilization of the stretch-shortening cycle in fast dynamic exercises such as the COD and plyometrics is thought to be similar in terms of the ability to change a muscle contraction from eccentric to concentric as rapidly as possible [11,36]. As such, training with different countermovement jumps has been utilized in several training interventions [20,35,37,38,39,40,41], inducing very small to very large effects [42]. The inconsistency in effects of the plyometric training interventions may be due to the lack of specificity towards the chosen COD test for performance measurements, as suggested in a review by Falch; Rædergård; van den Tillaar [36].

A common way of measuring COD performance in soccer players has been through the 505- agility test [43], measuring both total time and partial time (505-agility time), and is seen as a reliable measurement and increasing validity [44]. Although the 505- agility test is a common way of testing COD performance, it may not reflect improved physical abilities after a plyometric training intervention due to the specific requirements of a COD. Muscle activity is one of the factors directly contributing to performance in explosive dynamic movements [45], such as the COD and countermovement jumps in a horizontal and vertical direction. 

Earlier studies found inconsistent relationships between countermovement-jump and COD performance [11,46,47,48,49,50], varying by the tests assessed. Although electromyography (EMG) measurements have been assessed in COD [51,52,53,54] and countermovement-jumps [55,56], to the best of the authors knowledge, no earlier research has applied EMG when investigating the relationship of countermovement-jumps and COD. A high muscle peak activity is an indicator of muscular effort made during a particular action [57]. As such, it can serve as a tool for discovering exercises sharing the same neuromuscular characteristics found in COD. Consequently, this can develop muscular characteristics that strength and conditioning coaches perceive as lacking in their athletes with respect to COD.

Therefore, the aim of the present study was to investigate the relationship between jumping performance with COD performance together with examining the similarities and differences in peak muscle activation when utilizing different countermovement jumps with COD performance during a change of direction step.

Specificity is crucial when training to develop physical skills in soccer [5]. Thus, comparisons of performance and muscle activity may lead to more specific guidelines for future training interventions when utilizing different countermovement jumps to improve COD performance. It was hypothesized that the different countermovement jumps would match upon levels of peak muscle activity with COD and therefore these types of jumps could be used in plyometric training to enhance COD performance.

## 2. Materials and Methods

### 2.1. Method

A randomized controlled study with a within subject design was used to investigate the relationship of muscle activity in countermovement jumps and the pivoting step in a modified 505-agility test. 

### 2.2. Subjects

After contacting the coaching-staff of several local clubs seeking recruitment, twenty-one experienced soccer players (age: 21 ± 2.5 years, height: 182 ± 8 cm, body mass: 78 ± 13 kg, 2nd–6th national playing level) volunteered to participate in the study. Preferred kicking foot, hereby referred to as dominant foot, was the right foot for eighteen subjects and left for three of the subjects. The study complied with the current ethical regulations for research and approved by the Norwegian Center for Research Data project number: 42440, and conformed to the latest revision of the Declaration of Helsinki. The subjects were instructed not to consume alcohol and to avoid demanding physical activity twenty-four hours prior to testing.

### 2.3. Procedures

All subjects participated in a familiarization session in which they practised the modified 505-agility test and the different countermovement jumps to avoid a possible learning effect. Both the familiarization session and the test day started with a standardized warm-up, before the different countermovement jumps were performed, followed by the COD test. Subjects were tested one by one on the test day, with height and body mass being taken first, before the placement of electromyography (EMG) pads on ten muscles of the subjects’ dominant foot. Subsequently, a dynamic warm-up based on a protocol by Pagaduan; Pojskić; Užičanin; Babajić [58] was conducted, consisting of exercises such as butt kicks, carioca, high knees, reverse lunges, straight-leg march, power shuffle and jogging with squats. After the warm-up, the EMG sensors were connected to the pads, while reflecting markers were attached to different anatomical landmarks. The markers were used for kinematic analysis and used on the day of testing for controlling the technique when executing the different countermovement jumps. 

After all the equipment was attached, the subjects performed sub-maximal jumps for the different countermovement jumps as a part of the specific warm-up, before being tested for maximal performance in all jumps in a randomized order. The countermovement jumps consisted of jumping vertically for maximal height and horizontally for maximal length, performed bilaterally and unilaterally with the dominant foot. The subjects were instructed to jump as high or as far possible. The athletes’ hands were placed akimbo, to prevent the arms from contributing to jump performance, limiting the isolated effect of leg power [59]. At the unilateral jumps, the non-dominant foot was instructed to be kept passive and locked in a forward position throughout the jump, to prevent it from contributing to jump performance. Additionally, the subject needed to stand managing the landing for an attempt to be approved. Joint angles had to be approximately equal at 90 degrees in the knee joint for each jump condition, which was visually controlled by the research leader. The subjects rested for two minutes between each jump. The jumps executed with approved technique, displaying greatest height or length for each condition were used for further analysis. The horizontal jumps were performed on a soft rubber mat (Everroll, 8 mm, Regupol, Germany) and the jump length was measured manually with a measuring tape with an accuracy of 0.001 m. 

After finishing the jump tests, a re-warm-up for the COD started, which consisted of performing the test at sub-maximal intensities (50%, 70% and 90% of self-perceived maximum intensity). Pauses of one to two minutes were included between each run during the re-warm-up, where the athlete performed rotational movements from a protocol by Van den Tillaar; Lerberg; von Heimburg [60]. Then the COD test was performed with maximal intensity, with three minutes’ rest between each run. The COD test used was a modification of the 505-agility test, with a turn where the dominant foot performed the pivoting step, referred to as the COD step (left turn for right-foot-dominant athletes, the opposite for left-foot-dominant athletes). The set-up for the test was according to the test guidelines of Van Gelder; Bartz [61], starting and finishing the test by pushing a button placed upon a tripod.

### 2.4. Measurements

**Figure 1:**
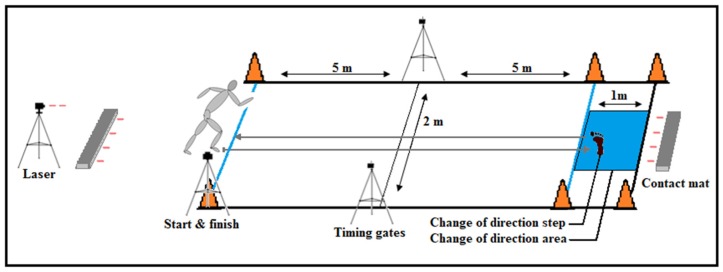
Set-up and dimensions for the change of direction (COD) test.

Total time to complete the COD test (10 m + COD + 10 m) and 505-agility time (5 m + COD + 5 m) were both measured. Total time was measured from when the subject manually pushed a button (Brower Timing Systems, Salt Lake Utah, USA, TS-T175) to start and stop the test, displaying total time on a wireless timer (Brower Timing Systems, Salt Lake Utah, USA, CM L5 MEM). The 505-agility time was measured by a wireless timing sensor (Ergotest Innovation, Porsgrunn, Norway) with a resolution of <0.01 s, sending and reflecting an infrared light beam. Both total and 505-agility time were used for statistical analysis. Peak velocity in the COD performance was found using a laser (CMP distance sensor, Noptel Oy, Uleåborg, Finland), placed on a tripod behind the starting position of the COD test, which was adjusted to point at the athlete’s pelvis while running towards the COD (Figure 1).

Muscle activity was measured using a wireless EMG with a sampling rate of 1 kHz (Ergotest Innovation, Porsgrunn, Norway) with electrodes (Zynex Neurodiagnostics, Englewood, CO, USA) on the muscles of the dominant foot. Before placing the electrodes, the skin was shaved and washed with alcohol. The electrode pads (11 mm contact diameter and 2 cm centre-to-centre distance) were placed along the presumed direction of the underlying muscle fibres on the lateral and medial vastii, rectus femoris, adductor longus, biceps femoris, semitendinosis, soleus, lateral gastrocnemius, gluteus medius and gluteus maximus muscle, according to the recommendations of Hermens; Freriks; Disselhorst-Klug; Rau [62]. The EMG raw signal was amplified and filtered using a preamplifier located as close as possible to the pickup point with the intention of minimizing the noise induced from external sources through the signal cables. The preamplifier had a common mode rejection ratio of 100 dB. The EMG raw signal was then bandpass-filtered (fourth-order Butterworth filter) with cut-off frequencies of 20 Hz and 500 Hz. The resulting EMG signals were converted to root mean square (RMS) signals. The highest observed EMG-signal prior take-off in the countermovement jumps and the step performing the COD turn were used for further analysis. Contact time in the COD step was found using a contact mat (Ergotest Innovation, Porsgrunn, Norway, IR-Contactmat-ML6TJP02- 870). The IR-contact mat sends and reflects an infrared carpet with a resolution of <2 ms, reflected by an IR-mirror, which detects contact when the infrared carpet is disrupted. 

**Figure 2:**
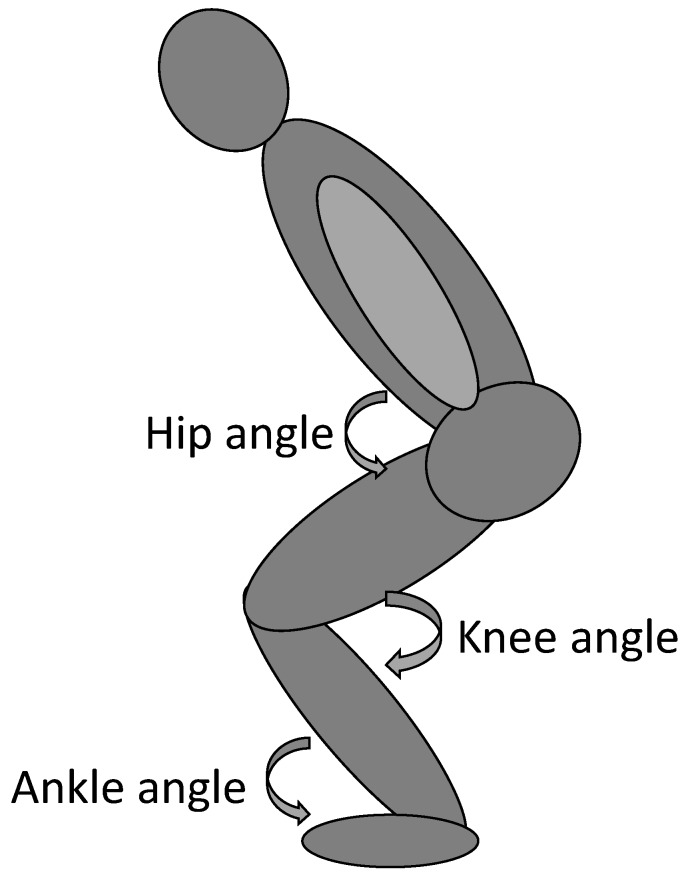
Joint angles in the countermovement jumps, retrieved from anatomical hallmarks.

The Qualisys Track Manager with a sample rate of 500 Hz (Qualisys Oqus, 8 cameras, Gothenburg, Sweden) was used for validating the technique of the countermovement jumps (Figure 2). Reflecting markers were placed on the following anatomical hallmarks: L5; acromion cluster (posterior, medial and lateral); C4; iliac crest; trochanter major; patella (lateral and medial) lateral and medial malleolus; tuber calcanei; and art. metatarsophalangeal. The reflecting markers create a biomechanical model. Data from the biomechanical model in the lowest depth of the countermovement was exported to Visual3D (Visual3D Professional v5.02.27, C-motion, Germantown, MD, USA) where kinematics was calculated and sagittal angle of the hip, knee and ankle joint was retrieved in which full hip and knee extension and plantar flexion was 180°. All the equipment used for the countermovement jumps and COD test were synchronized in Musclelab V.18 (Musclelab, Ergotest Innovation, Porsgrunn, Norway).

### 2.5. Statistical Analysis

Statistical analysis was conducted in SPSS V. 25 (SPSS, Inc., Chicago, IL, USA). Descriptive statistics are presented as mean ± standard deviation. The EMG and kinematic data were analysed by a one-way analysis of variance (ANOVA) with repeated measures. The Holm–Bonferroni test was conducted post hoc when significant differences were observed. Violations of the assumption of sphericity was corrected for by the Greenhouse−Geisser correction. The effect of the different conditions upon muscle activity was presented as eta squared (η^2^) where 0.01 < η^2^ < 0.06 constituted a small effect, 0.06 < η^2^ < 0.14 a medium effect, and η^2^ > 0.14 a large effect [63]. Effect size (ES) of pairwise comparisons were calculated according to Cohen’s d, and interpretations of the magnitude were as follows: 0–0.2 = trivial, 0.2–0.5 = small, 0.5–0.8 = medium, >0.8 = large [63]. Correlational analysis was conducted by Pearson’s *r*. The alpha-level was set at *p* < 0.05.

## 3. Results

Significant differences in knee and hip joint angles were found between different countermovement jumps (F ≥ 3.84; p < 0.04; η*^2^* ≥ 0.49). Post hoc tests revealed significant differences between knee joint angle when performing the horizontal unilateral jump, compared to horizontal bilateral and vertical unilateral jumps (*p* < 0.01; ES ≥ 1.5). The hip joint was statistically significantly different between all jumps (*p* < 0.05; ES ≥ 0.50), except when comparing the unilateral jumps (*p* = 0.93; ES = 0.02; Table 1).

The average performances of the different jumps and COD variables are shown in Table 2.

**Figure 3:**
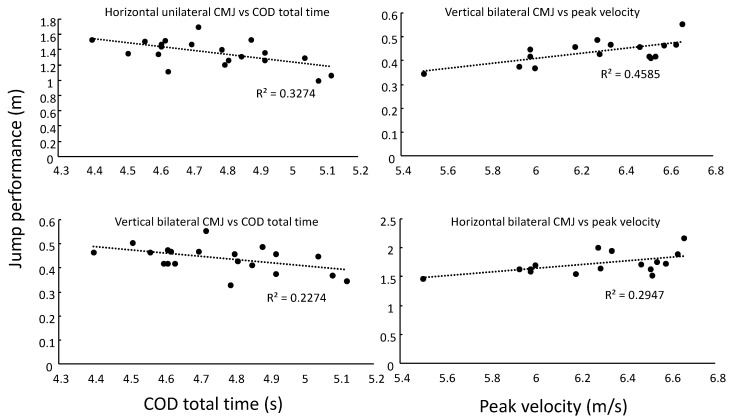
Correlations of jump performance with total time to complete the COD test and peak velocity.

Peak velocity, 505-agility time and total time to complete the COD test were all significantly correlated with each other (*r* > −0.57, *p* ≤ 0.03). Performance in the vertical bilateral (*r* = −0.48, *p* = 0.03) and horizontal unilateral jumps (*r* = −0.57, *p* = 0.03) were found to be significantly correlated with total time to complete the COD test, in which an increase in jump height and jump length related with a decrease in total time COD test. Both bilateral jumps were significantly correlated with peak velocity (*r* > 0.54, *p* ≤ 0.03); increase in jump height and jump length related with an increase in peak velocity (Figure 3). None of the jumps were significantly correlated with the 505-agility time nor contact time in the COD step (*r* < −0.35, p ≥ 0.2) (Table 3). 

**Figure 4:**
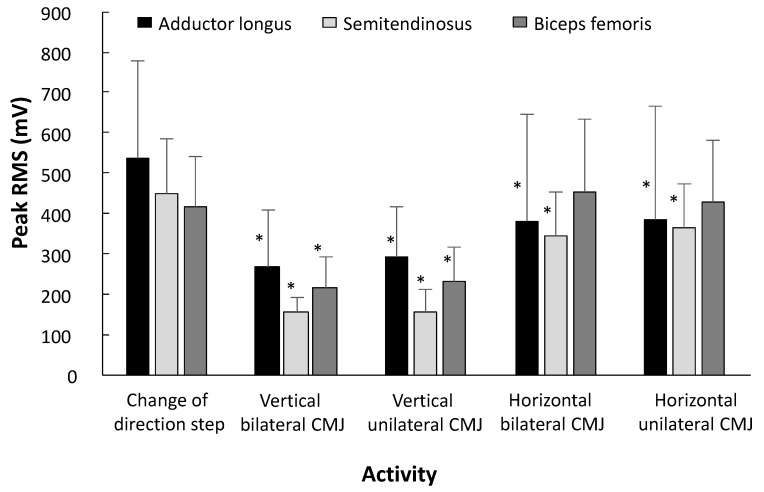
Comparison of peak (±SEM) muscle activity in the change of direction step (COD) and the different countermovement jumps. CMJ = countermovement jump. * indicates a significant difference in muscle activity compared with the COD step, on a *p* < 0.05 level.

Most muscles had the same peak activation between the different jumps with the COD step (Table 4). Only significant different muscle activities were observed for the adductor longus, semitendinosus and biceps femoris (F ≥ 7.9; p < 0.02; η*^2^* ≥ 0.56). Post hoc tests revealed that all countermovement jumps had statistically significant lower muscle activity compared to the COD step (p ≤ 0.047, ES ≥ 0.70), except not for biceps femoris activity (p ≥ 0.6, ES ≥ 0.10) during the horizontal jump and the COD step (Table 4 and Figure 4). 

## 4. Discussion

The aim was to investigate the relationship between jumping performance with COD performance together with examining the similarities and differences in peak muscle activation when utilizing different countermovement jumps with COD performance during a change of direction step. Greater knowledge of the relationship in performance and muscle activation between countermovement jumps and COD may lead to improved specific guidelines for future training interventions. The main findings were that both performance in vertical bilateral and horizontal unilateral countermovement jumps were related to COD performance (Table 2). The only differences in muscle activation when comparing the countermovement jumps with the COD step were observed in the adductor longus, biceps femoris and semitendinosus. 

Several studies support the finding that countermovement jumps share physical similarities with COD [11,47,49]. Like Castillo–Rodríguez; Fernández–García; and Chinchilla–Minguet; Carnero [11] observed a high correlation with the countermovement jump (*r* = 0.6) when using a similar sprint with a 180° turn to measure COD performance, supporting the findings of the current study that also found The relationship observed might be due to a similar dependency on reactive strength [46] and peak muscle activities. The unilateral vertical jump revealed only a small relationship with COD, possibly limited by balance and coordination affecting the net forces produced, which is an important aspect of the turn in a COD [64]. The COD requires production of both vertical and horizontal ground reaction forces [10], where the 180° turn is often performed bilaterally [65]. The observed correlations indicate limitations of the task specific movement of the unilateral vertical countermovement jump in relation to COD, due to neither being performed bilaterally nor producing horizontal forces.

The current study measured 505-agility time as well, which did not correlate with performance in any of the countermovement jumps. As such, the correlation observed between jump performance and COD performance may be largely influenced by the straight line sprint, not the actual ability to decelerate and re-accelerate in a COD [66,67]. The measurement of peak velocity supported this finding, as peak velocity was related to the total and 505-agility times in the COD test, plus performances in the bilateral jumps (Table 2). Thus, athletes performing better in bilateral countermovement jumps may be better sprinters, which may explain why COD total time was related to jump performance, but not 505-agility time. This assumption is reasonable, since the relationship between jump and sprint performance is well known [68,69,70,71] and increases at longer sprints [49].

The 180-degree turn requires the athlete to complete the change in momentum to the opposite direction, not allowing for velocity maintenance [72]. In addition, to effectively change momentum as rapidly as possible, athletes rotate their trunk towards the desired direction of travel prior to the COD step [73]. This rotation may explain the high peak muscle activity of the adductor longus in the COD, as the adductors function as stabilizers of the hip in CODs [54,74]. To change momentum, the athletes need to decelerate by maximizing ground contact time whereby the muscles eccentrically decelerate joint movements [75]. In countermovement jumps, there is small length-changes in the adductor longus which produces little mechanical work [76]. As the adductor longus primary objective is to adduct the hip in the frontal plane, it contributes minimal for force production in the sagittal plane, in which the counter-movement jumps were performed. At least when compared to the COD step, where the adductors contribute to the hip adduction movement at the start and end of the stance [77]. 

The hamstrings also revealed higher muscle activity in the COD step compared to the countermovement jumps (Figure 4). In the turn of a COD, the hamstrings works eccentrically to control the knee flexion [46] in the COD manoeuvre by decelerating knee joint moments, controlling the load upon the knee joint [54,78,79]. Since the turn of the COD test conducted required a complete change in momentum, great eccentric forces are required of the hamstrings to decelerate. 

However, muscle activity of the biceps femoris was similar in the COD step as in the horizontal jumps (Figure 4). The high peak muscle activity observed in the biceps femoris for the horizontal jumps might be to produce great forces in the horizontal axis, with the hip further away from, and behind, the centre of mass [80,81]. Horizontal jumps have been shown to induce slower eccentric stretch displacement and time over which force is applied [80]. Furthermore, Fukashiro, Besier, Barrett, Cochrane, Nagano; and Lloyd [80] suggested that force applied over a greater distance in horizontal jumps, utilizing a slower stretch-shortening cycle, is beneficial, allowing greater forces produced by the biceps femoris. The opposite accounts for the vertical jumps, which displayed low muscle activity in both semitendinosus and biceps femoris, compared to muscle activity in the COD. In vertical jumps, early activation of the biceps femoris has been found to negatively influence the joint power transfer [80], reducing the effect of the stretch-shortening cycle, which is a key factor for performance in vertical jumps [82]. As such, the low muscle activity of the hamstrings in vertical jumps could be a result of promoting a fast stretch-shortening cycle. The importance of producing forces by the hip flexors, knee extensors and plantar flexors has been addressed for both COD [72,73,74,75] and the countermovement jump [80,81,83,84]. The muscle activation observed indicated similarities in the required force production by the lower-limb muscles when comparing the COD step with countermovement jumps. The COD and countermovement jumps were only separated by the muscle activity of the adductor longus, semitendinosus and biceps femoris.

However, limitations of the jumps in the current study must be addressed. Technique in the countermovement jumps was attempted to be visually controlled for by practice, although this was only partially successful (Table 1). In addition, the COD test consisted of only one 180° turn. Correlation of countermovement jumps and 505- agility times may be greater in CODs performed at smaller degrees such as a 90- or 45-degree turn, where the athlete can maintain velocity and transfer momentum and other muscle activities may be required. Another limitation of the study is that forces in the COD step and countermovement jumps were not measured. Future studies should include CODs with difference degrees of turn and a force plate to give more information about comparisons of muscle activity and forces produced in the COD step with other degrees of turn with these countermovement jumps. 

## 5. Conclusions

The countermovement jump performances (vertical bilateral and horizontal unilateral) are related with completion time for the COD test, but none of the jumps correlated significantly with 505-agility time. Peak muscle activity of most muscles are similar between the jumps and the COD step, indicating similar muscular demands between these activities. However, higher adductor longus and hamstring activities are required to respectively stabilize the hip and decelerate knee joint movements when turning in a COD compared with the jumps. Based on the findings of the present study, we suggest athletes and strength and conditioning coaches to include vertical bilateral and horizontal unilateral jumps in their practice to investigate if training these exercises enhances 180° COD performance as these correlate positively. However, when there are weaknesses in the adductor longus and/or hamstring muscles, other exercises than the jumps studied in the present study, should be performed, since these did not reach comparable muscles activation levels with 180° CODs. 

## Figures and Tables

**Table 1:** Descriptive statistics for the peak joint angles at deepest position when performing the different countermovement jumps.

Joint Angle	Vertical Unilateral	Vertical Bilateral	Horizontal Unilateral	Horizontal Bilateral
Ankle (°)	67.9 ± 2	67.8 ± 1.3	67.9 ± 6.5	66.6 ± 2.4
Knee (°)	92.1 ± 5.2 ‡	91.6 ± 6	88 ± 5.4	92.2 ± 3.4 ‡
Hip (°)	68.1 ± 14.6 *	83.8 ± 15.2	68.5 ± 16.7 *	78.2 ± 9.8

* Indicates a significant difference with all bilateral jumps, on a *p* < 0.05 level; ‡ indicates a significant difference with the horizontal unilateral jump, on *p* < 0.05 level.

**Table 2:** Descriptive statistics of performance in the change of direction- and counter-movement tests.

Change of Direction Performances		Countermovement Jump Performance	
COD total time (s)	4.8 ± 0.2	Vertical bilateral (m)	0.445 ± 0.05
505-agility time (s)	2.6 ± 0.1	Vertical unilateral (m)	0.275 ± 0.03
Peak velocity (m/s)	6.3 ± 0.3	Horizontal bilateral (m)	1.76 ± 0.21
Contact time (s)	1.4 ± 0.2	Horizontal unilateral (m)	1.38 ± 0.17

**Table 3:** Correlation of performance variables in the change of direction (COD) test and performance in the counter movement jumps (CMJ).

	Change of Direction Test			Counter Movement Jump			
Variable	505-Agility Time	Contact Time	Peak Velocity	Vertical Bilateral	Vertical Unilateral	Horizontal Bilateral	Horizontal Unilateral
COD total time	0.837 *	−0.172	−0.711 *	−0.476 *	−0.256	−0.434	−0.572 *
505-agility time		−0.030	−0.569 *	−0.099	−0.295	−0.163	−0.349
Contact time			−0.371	−0.114	0.020	−0.185	0.042
Peak approach velocity				0.676 *	0.425	0.540 *	0.476
Vertical bilateral CMJ					0.601 *	0.720 *	0.624 *
Vertical unilateral CMJ						0.448 *	0.505 *
Horizontal bilateral CMJ							0.586 *

* Indicates a significant correlation at the 0.05 level.

**Table 4:** Peak (±SD) EMG activity for the different muscles during the change of direction step and the different countermovement jump.

Muscles	Change of Direction	Vertical Bilateral	Vertical Unilateral	Horizontal Bilateral	Horizontal Unilateral
Gluteus maximus	337 ± 163	224 ± 184	227 ± 179	304 ± 301	287 ± 229
Gluteus medius	442 ± 166	350 ± 374	401 ± 345	622 ± 820	486 ± 428
Adductor longus	536 ± 241	270 ± 139 *	292 ± 125 *	382 ± 263 *	384 ± 284 *
Semi-tendinosus	451 ± 133	154 ± 37 *	156 ± 57 *	343 ± 110 *	367 ± 107 *
Biceps femoris	416 ± 124	218 ± 74 *	233 ± 83 *	454 ± 179	429 ± 152
Vastus lateralis	798 ± 312	718 ± 291	759 ± 370	753 ± 485	844 ± 564
Rectus femoris	481 ± 114	443 ± 133	455 ± 133	447 ± 345	473 ± 241
Vastus medialis	749 ± 435	823 ± 368	783 ± 444	809 ± 583	905 ± 581
Gastrocnemius	497 ± 170	387 ± 106	381 ± 125	533 ± 348	518 ± 364
Soleus	757 ± 607	249 ± 136	292 ± 186	309 ± 243	346 ± 248

* indicates a significant difference with the change of direction step on a *p* < 0.05 level.
